# Application of dual-stream 3D convolutional neural network based on ^18^F-FDG PET/CT in distinguishing benign and invasive adenocarcinoma in ground-glass lung nodules

**DOI:** 10.1186/s40658-021-00423-1

**Published:** 2021-11-02

**Authors:** Xiaonan Shao, Rong Niu, Xiaoliang Shao, Jianxiong Gao, Yunmei Shi, Zhenxing Jiang, Yuetao Wang

**Affiliations:** 1 Department of Nuclear Medicine, The Third Affiliated Hospital of Soochow University, Changzhou, 213003 China; 2 Changzhou Key Laboratory of Molecular Imaging, Changzhou, 213003 China; 3 Department of Radiology, The Third Affiliated Hospital of Soochow University, Changzhou, 213003 China

**Keywords:** Lung adenocarcinoma, Differential diagnosis, Deep learning, Fluorodeoxyglucose F18, Positron emission tomography-computed tomography

## Abstract

**Purpose:**

This work aims to train, validate, and test a dual-stream three-dimensional convolutional neural network (3D-CNN) based on fluorine 18 (^18^F)-fluorodeoxyglucose (FDG) PET/CT to distinguish benign lesions and invasive adenocarcinoma (IAC) in ground-glass nodules (GGNs).

**Methods:**

We retrospectively analyzed patients with suspicious GGNs who underwent ^18^F-FDG PET/CT in our hospital from November 2011 to November 2020. The patients with benign lesions or IAC were selected for this study. According to the ratio of 7:3, the data were randomly divided into training data and testing data. Partial image feature extraction software was used to segment PET and CT images, and the training data after using the data augmentation were used for the training and validation (fivefold cross-validation) of the three CNNs (PET, CT, and PET/CT networks).

**Results:**

A total of 23 benign nodules and 92 IAC nodules from 106 patients were included in this study. In the training set, the performance of PET network (accuracy, sensitivity, and specificity of 0.92 ± 0.02, 0.97 ± 0.03, and 0.76 ± 0.15) was better than the CT network (accuracy, sensitivity, and specificity of 0.84 ± 0.03, 0.90 ± 0.07, and 0.62 ± 0.16) (especially accuracy was significant, *P*-value was 0.001); in the testing set, the performance of both networks declined. However, the accuracy and sensitivity of PET network were still higher than that of CT network (0.76 vs. 0.67; 0.85 vs. 0.70). For dual-stream PET/CT network, its performance was almost the same as PET network in the training set (*P*-value was 0.372–1.000), while in the testing set, although its performance decreased, the accuracy and sensitivity (0.85 and 0.96) were still higher than both CT and PET networks. Moreover, the accuracy of PET/CT network was higher than two nuclear medicine physicians [physician 1 (3-year experience): 0.70 and physician 2 (10-year experience): 0.73].

**Conclusion:**

The 3D-CNN based on ^18^F-FDG PET/CT can be used to distinguish benign lesions and IAC in GGNs, and the performance is better when both CT and PET images are used together.

**Supplementary Information:**

The online version contains supplementary material available at 10.1186/s40658-021-00423-1.

## Introduction

With the application of low-dose CT (LDCT) and the screening of COVID-19, the detection rate of early lung adenocarcinoma manifested as ground-glass opacity nodule (GGN) has increased rapidly [1, 2]. To treat lung GGN, an important early step is to estimate the probability of malignancy. More aggressive treatment methods should be considered if the predicted probability of malignancy is high [3]. GGNs are associated with various lung diseases, such as inflammatory pseudotumor, tuberculoma, sclerosing hemangioma, lymphoepithelioma, and non-small cell lung cancer (NSCLC) [4]. The imaging features used to determine lesion malignancy include size, density, follow-up stability, edge appearance, wall thickness, and the presence of cavitation and calcification [5–7]. The clinical management of GGN is determined based on the assessed risk, which may involve routine CT follow-up, functional imaging, and/or tissue biopsy [5, 8, 9].

Several studies [10, 11] found that a single CT morphological feature and quantitative parameter did not have a good diagnostic value for GGN. In recent years, the extraction of quantitative imaging features from medical scans ("Radiomics" [12–14]) has attracted broad research interest, and it has been considered as a possible method for distinguishing benign and malignant lung nodules [15]. The basic principle of radiomics is to use image information that may be clinically relevant but not noticed by human eyes [16]. Radiomics can perform a comprehensive analysis of the region of interest, while the biopsy can only capture a small part of the lesion [17]. Radiomics has been extensively studied in PET/CT imaging [18]. The latest study from Palumbo et al. [19] found that the shape and texture features of ^18^F-FDG PET/CT could provide more information for distinguishing benign and malignant lung nodules than conventional imaging functions alone.

Machine learning methods have been introduced into medical image analysis and evolved into deep learning methods (especially the use of multilayer convolutional neural networks (CNNs)) [20–24]. Tau et al. [25] showed that using CNN to analyze the primary tumors of newly diagnosed NSCLC patients with segmented PET images could make reasonable predictions for N category. Another study found that it was feasible to use CNN to automatically locate and classify the ^18^F-FDG PET uptake patterns in foci suspicious and non-suspicious for cancer in patients with lung cancer and lymphoma; moreover, it could achieve higher diagnostic performance when using CT and PET images at the same time [26]. As far as we know, three-dimensional convolutional neural networks (3D-CNN) are often used to segment tumors in PET/CT images [27, 28], but the dual-stream CNN (data obtained from PET and CT as inputs) [24] has not been used in the study of benign and malignant differentiation of GGN.

Since the clinical management strategies for treating benign lesions and invasive adenocarcinoma (IAC) are completely different [9], the accurate identification of benign and malignant GGN is of great importance. Therefore, this study aims to train, validate, and test the 3D-CNN based on ^18^F-FDG PET/CT images and evaluate CT, PET, and PET/CT 3D-CNN performance in distinguishing benign lesions and IAC.

## Materials and methods

### Participants

**Fig. 1:**
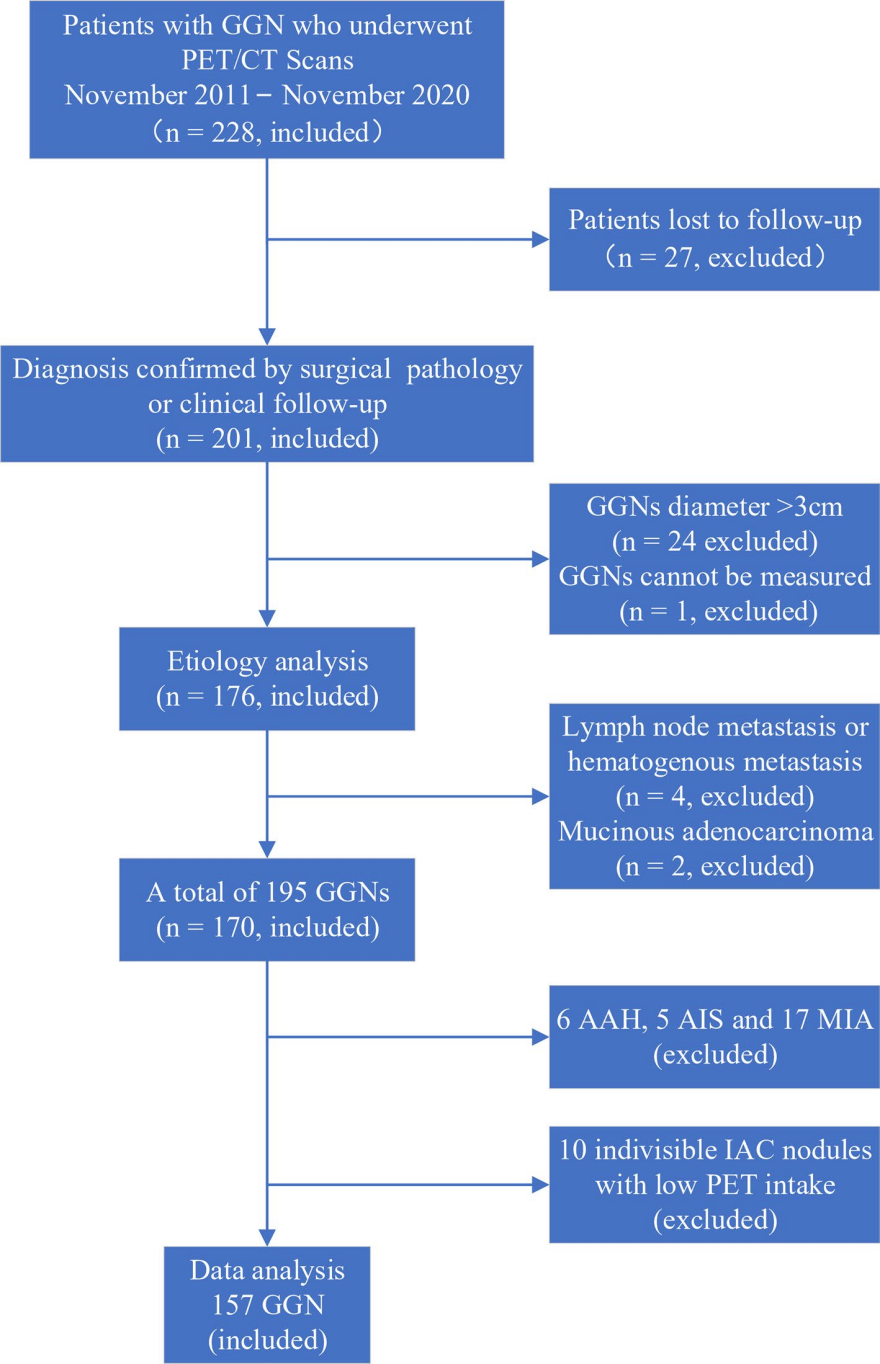
Study flowchart. GGN, ground-glass nodule; AAH, atypical adenomatous hyperplasia; AIS, adenocarcinoma in situ; MIA, minimally invasive adenocarcinoma

In this single-center study, we reviewed 228 GGN patients who underwent ^18^F-FDG PET/CT examinations in our hospital from November 2011 to November 2020. The Institutional Ethics Committee reviewed the retrospective analysis (No. [2020] KD 075) and agreed that the written informed consent was not required. The inclusion criteria for patients were as follows: (1) patients underwent therapeutic surgical resection within one month after PET/CT examination and the postoperative pathological data were complete (pathologically confirmed), or the volume of benign GGN decreased during CT follow-up (clinical confirmed); (2) maximum GGN diameter ≤ 30 mm. The exclusion criteria were as follows: (1) lung adenocarcinoma stage > IA; (2) poor image quality or low FDG uptake, and the lesion was difficult to measure; (3) history of malignant tumors in the past 5 years; (4) severe liver disease or diabetes; (5) other pathological subtypes: atypical adenoma hyperplasia (AAH), adenocarcinoma in situ (AIS) or microinvasive adenocarcinoma (MIA). The data of included patients were collected by reviewing the cases or follow-up by telephone. The patient selection process is shown in Fig. 1.

### FDG PET/CT image acquisition

The image acquisition protocol was based on the Imaging Biomarker Standardization Initiative (IBSI) Reporting Guide [29]. The details of all procedures are described in Additional file 1. Within one month before surgery, the patient received ^18^F-FDG PET/CT examination (Biograph mCT 64, Siemens, Erlangen, Germany). According to the European Association for Nuclear Medicine (EANM) guideline 1.0 (version 2.0 was released in February 2015) [30], ^18^F-FDG PET/CT images were acquired at 60 ± 5 min after ^18^F-FDG injection. All PET/CT images were reconstructed on the processing workstation (TrueD software, Siemens Healthcare). CT data were used to perform attenuation correction on PET images, and the corrected PET image was combined with CT image. Respiratory-gated technology was not used in the acquisition process.

### Image preprocessing

**Fig. 2:**
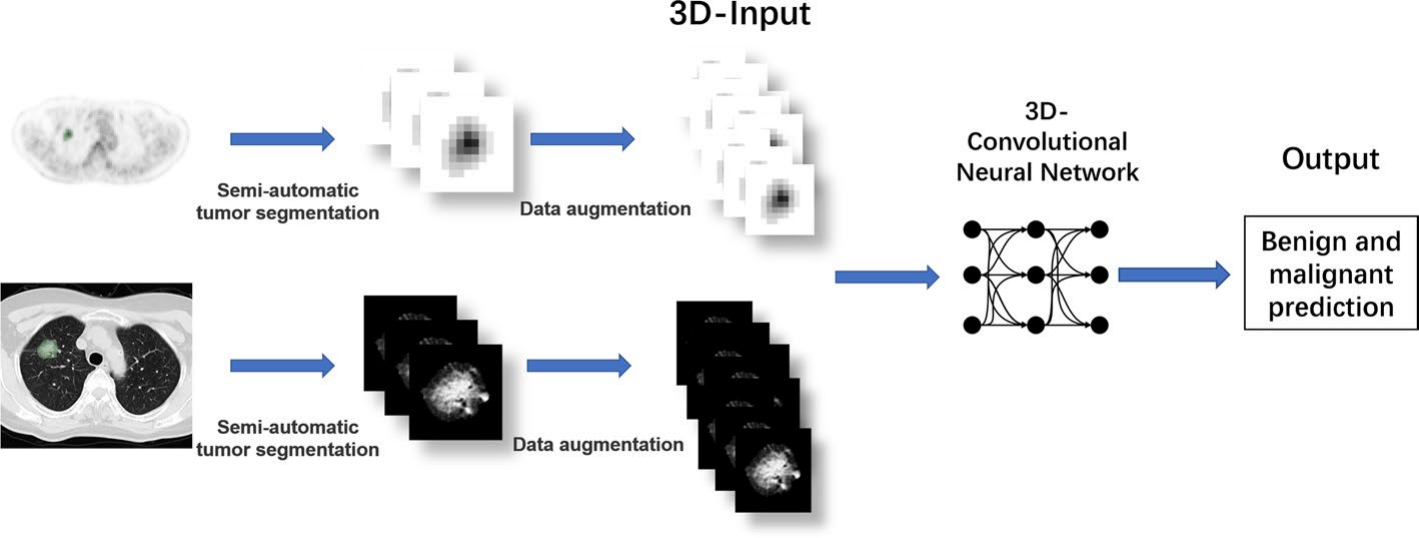
The overview of the process of generating deep learning algorithms to predict benign and malignant GGNs

Figure 2 summarizes the overall approach to developing a deep learning model. All images were segmented using 3D-Slicer (version 4.11.20200930, www.slicer.com). For PET images, we used a semiautomatic segmentation method developed by Beichel et al. [31]. To consider the pattern of tumor edge, the generated boundary was dilated by one pixel. To eliminate the influence of lung background noise, we set the outside area of lesion boundary as a value 0. Since the sample size was small, to ensure the learning effect, simplify the task, and reduce the memory requirements and processing time, we segmented a 3D volume that occupied a small part of the entire image. This 3D volume was used as the input for PET model. Because the matrix size of FDG PET varied according to the reconstruction algorithm, the nearest neighbor interpolation method was used to reslice the segmented cube-shaped volume into isotropic spacing (for example, 4.07 mm^3^).

For CT images (3 mm), we used NVIDIA AI-Assisted Annotation (3D-Slicer built-in) and the boundary-based CT segmentation models to process lung nodule images. To consider the pattern of tumor edge, the generated boundary was dilated by 3.00 mm. To eliminate the influence of lung background noise, we filled the outside area of lesion boundary with a value -2000 (all values were 0 after normalization). Similarly, we segmented a 3D volume that occupied a small part of the entire image. This 3D volume was used as the input for CT model. Since the size of CT matrix varied, the nearest neighbor interpolation method was used to reslice the segmented cube-shaped volume into isotropic spacing (for example, 0.73mm^3^).

### The training of deep learning model

Due to the limited size of training data, we used a rotation method for data augmentation. More specifically, image augmentation aimed to develop a robust deep learning model against the rotation of tumors, which can be affected by position and location of tumors. We rotated the segmented tumor for each image in the training data by 90°, 180°, and 270°. This type of image augmentation is usually used in deep learning training for natural images and medical images [32, 33].

The segmented tumor was the deep learning model's input whose architecture was based on 3D-CNN [34]. FDG-PET was converted to SUVbw (bodyweight units), and the SUV values ranging from [0–15] were linearly mapped to a [0–1] range. Similarly, CT images were expressed in Hounsfield units (HU), and HU values ranging from [− 1000–400] were linearly mapped to a [0–1] range. Since the sizes of input images were different, CNN was designed to generate output regardless of the input matrix size.

Due to the lack of available data, we adopted a cross-validation method [35] to evaluate the model reliably. The fivefold cross-validation was applied for the training data, and the testing data were used until the model was optimized by the training set/internal validation set of the cross-validation (based on maximum AUC). To train the model, the Adam optimizer (initial learning rate 0.0001) was used. The number of epochs for iterative training was set as 50. For comparison, we constructed three 3D-CNNs: CT, PET, and PET/CT. Additional file 1 provides detailed ^18^F-FDG PET/CT image acquisition methods, tumor segmentation, and deep learning model generation.

### Statistical analysis

Propensity score matching (PSM) was used to identify a group of GGNs with similar baseline characteristics. The propensity score was the conditional probability of a specific exposure given a set of covariates measured at baseline. The non-parsimonious multivariate logistic regression model was used to estimate the propensity score, with benign and malignant as grouping variables, and all the baseline characteristics listed in Table 1 are covariates. The greedy-matching algorithm was used to generate 1:4 matching pairs with replacement. Since the sample size was small, the difference in propensity score between the two groups was set within 0.6 [36]. The specific parameters are listed in Additional file 1.

**Table 1:** Comparison of baseline characteristics of the two GGN groups after PSM

Variables	Benign	IAC	Standardized diff	***P***-value
***N***	23	92		
Age (years)	57.83 ± 10.90	59.16 ± 8.62	0.1361	0.530
Fasting blood glucose	6.93 ± 1.87	6.73 ± 1.71	0.1124	0.621
*Gender*			0.3991	0.147
Female	9 (39.1)	54 (58.7)		
Male	14 (60.9)	38 (41.3)		
*Smoking history*			0.3714	0.168
No	13 (56.5)	68 (73.9)		
Yes	10 (43.5)	24 (26.1)		
*GGN number grouping*			0.0967	0.875
Solitary	16 (69.6)	68 (73.9)		
Multifocal	7 (30.4)	24 (26.1)		

The results in the table are expressed as Mean ± SD/*N* (%)

GGN, ground-glass nodule; PSM, propensity score matching; IAC, invasive adenocarcinoma

The continuous variables were expressed as mean (standard deviation) (Gaussian distribution) or median (range) (skewed distribution), and categorical variables were expressed as number or proportion. *χ*^2^ (categorical variables), Student’s *t* test (normal distribution), or Mann–Whitney U test (skewed distribution) was used to detect the differences between benign and malignant groups (binary variable). All the analyses were performed using the statistical software packages R (http://www.R-project.org, The R Foundation) and EmpowerStats (http://www.empowerstats.com, X&Y Solutions, Inc, Boston, MA). *P* values less than 0.05 (two-sided) were considered statistically significant.

We used different CNNs to analyze the data, and the area under the receiver operating characteristic curve (AUC), accuracy, sensitivity, specificity, positive predictive value (PPV), and negative predictive value (NPV) were calculated to evaluate the performance of different CNNs on the training set and testing set.

## Results

A total of 157 GGNs were included in the final data analysis. Among them, 23 were benign (including four fungal infections, one tuberculosis, four granulomatous inflammation, two organizing pneumonia, one alveolar epithelial bronchiolar metaplasia, and 11 with other inflammatory lesions), and 134 were IAC. The comparison of baseline characteristics between the two GGN groups after PSM is shown in Table 1.

Twenty-three benign nodules (maximum diameter 16.4 ± 6.6 mm; range 4.5–28.5 mm) and 92 IAC nodules (maximum diameter 19.4 ± 6.5 mm; range 5.5–30.0 mm) were included in the study. These 115 nodules were from 106 patients, including 46 males and 60 females. The average age was 59.2 ± 9.1 years old (range 31–78 years old), and the fasting blood glucose was 6.8 ± 1.7 mmol/L (range 4.1–11.0 mmol/L). There were 29 smokers (27.4%), and 22 cases (20.8%) had multifocal GGNs. We used stratified random sampling to divide the data set (*n* = 115) into training data (*n*= 82; IAC = 65, benign = 17) and testing data (*n * = 33; IAC = 27, benign = 6) according to a 7:3 ratio. There was no overlap between the two sets.

**Fig. 3:**
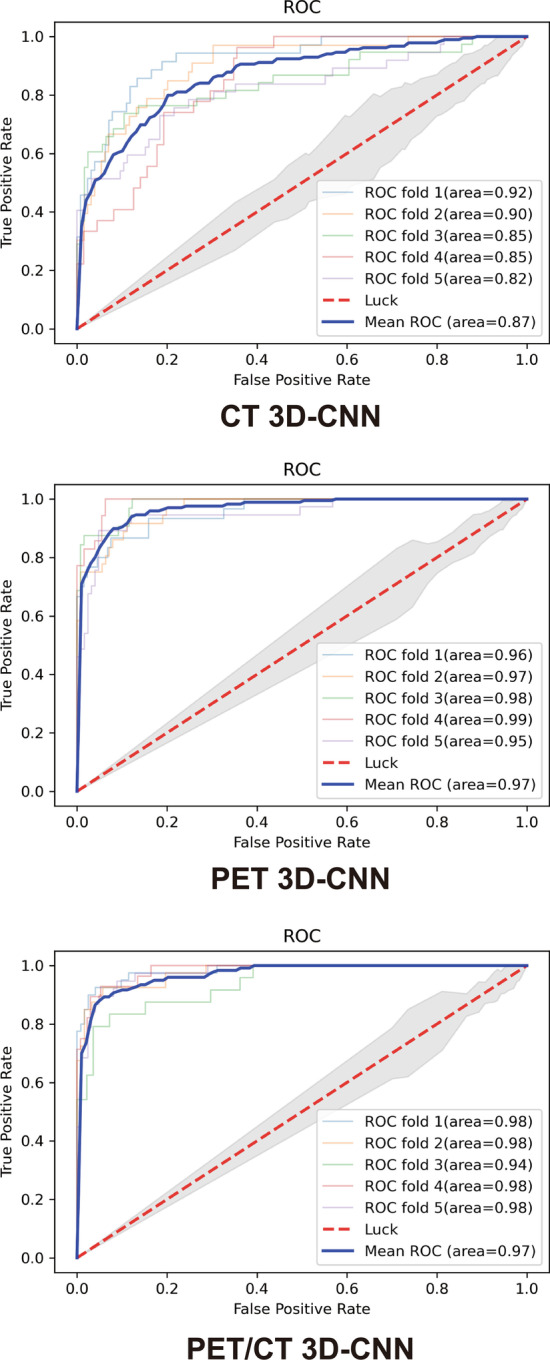
The ROC of three 3D-CNNs after fivefold cross-validation

After the three 3D-CNNs were cross-validated (fivefold cross-validation) on augmented training data, they were used to predict GGN malignancy (IAC vs. benign) on the testing set. The average AUC of the CT network in the training set was 0.87 ± 0.04, the average accuracy was 0.84 ± 0.03, and the average sensitivity and specificity were 0.90 ± 0.07 and 0.62 ± 0.16; in the testing set, the accuracy, sensitivity, and specificity were 0.67, 0.70, and 0.50 (Fig. 3 and Table 2).

**Table 2:** The performance indicators of three 3D-CNNs

Model	Accuracy	Sensitivity	Specificity	PPV	NPV
*Training set*					
CT 3D-CNN	0.84 ± 0.03	0.90 ± 0.07	0.62 ± 0.16	0.90 ± 0.04	0.63 ± 0.15
PET 3D-CNN	0.92 ± 0.02	0.97 ± 0.03	0.76 ± 0.15	0.94 ± 0.04	0.88 ± 0.09
PET/CT 3D-CNN	0.93 ± 0.01	0.98 ± 0.01	0.76 ± 0.06	0.94 ± 0.02	0.90 ± 0.09
*Testing set*					
CT 3D-CNN	0.67	0.70	0.50	0.86	0.27
PET 3D-CNN	0.76	0.85	0.33	0.85	0.33
PET/CT 3D-CNN	0.85	0.96	0.33	0.87	0.67

The results in the table are expressed as mean ± SD

3D-CNN, three-dimensional convolutional neural network; PPV, positive predictive value; NPV, negative predictive value

The average AUC of PET network in the training set was 0.97 ± 0.02, the average accuracy was 0.92 ± 0.02, the average sensitivity and specificity were 0.97 ± 0.03 and 0.76 ± 0.15; in the test set, the accuracy, sensitivity, and specificity were 0.76, 0.85, and 0.33 (Fig. 3 and Table 2). These results showed that, in the training set, the average AUC and various performance indicators of PET network were better than those of CT network (especially AUC, accuracy, and NPV were significant, *P*-value was 0.003, 0.001, and 0.013, respectively); in the testing set, the performance indicators of both CT and PET networks declined (especially the specificity was 0.50 and 0.33, respectively); however, when the PPV was similar, the accuracy, sensitivity, and NPV of the PET network were still higher than those of CT network.

The average AUC of the dual-stream PET/CT network in the training set was 0.97 ± 0.02, the average accuracy was 0.93 ± 0.01, the average sensitivity and specificity were 0.98 ± 0.01 and 0.76 ± 0.06; in the testing set, the accuracy, sensitivity, and specificity were 0.85, 0.96, and 0.33 (Fig. 3 and Table 2). In the training set, the average AUC and various performance indicators of the PET/CT network were almost the same as the PET network (*P*-value was 0.372–1.000) and were better than the CT network (especially AUC, accuracy, sensitivity, and NPV were significant, *P*-value was 0.003, < 0.001, 0.043, and 0.010, respectively); in the testing set, the performance indicators of PET/CT network declined (especially the specificity was 0.33); however, when the PPV was similar, the accuracy, sensitivity, and NPV of PET/CT network were still higher than both CT and PET networks.

To further test and evaluate the performance of dual-stream PET/CT network, we used the testing set to compare the accuracy of two nuclear medicine physicians with the PET/CT network. The results showed that the accuracy of PET/CT network was higher than the two nuclear medicine physicians [0.85 vs. Physician 1 (3-year experience): 0.70 vs. Physician 2 (10-year experience): 0.73].

## Discussion

In this study, we developed a dual-stream 3D-CNN that can distinguish between benign lesions and IAC based on clinical ^18^F-FDG PET/CT GGN images. In the testing set, the accuracy and sensitivity of PET/CT network were 0.85 and 0.96, which were comparable to or higher than those of the CT and PET networks. Moreover, compared with the diagnosis results of two nuclear medicine physicians, PET/CT network also showed better performance.

The 2017 Fleischner Association Guidelines [9] recommend that the planned CT follow-ups be carried out for GGNs, and persisted GGNs can be determined according to the dynamic changes of the nodules; for example, the infectious or inflammatory lesions may shrink or disappear during follow-up. However, long-term CT follow-up brings severe anxiety and repeated radiation exposure to patients, not applicable to some patients. Therefore, more effective imaging techniques or the construction of multifactor predictive models are needed to distinguish between benign and malignant GGNs. Among the many models, only the Brock model [37] considers the characteristics of GGNs and adjusts the correlation coefficient according to the nodule type. However, this model's application is complicated, and its modeling data came from the initially screened patients whose malignancy rate was low (5.5%). Although, in recent years, the focus of CT radiomics has turned to the field of GGN invasiveness [38], the problem of distinguishing benign and malignant GGNs is still unsolved. Gong et al. [39] developed a radiomics feature analysis method based on CT images to distinguish benign and malignant GGNs and confirmed its feasibility. The AUC of identifying benign and IAC nodules in the training set was 0.93, and the accuracy in the testing set was 0.61, which was higher than the two radiologists (0.53 and 0.56, respectively) [39]. In this study, the average AUC of CT network also reached 0.87, and the accuracy was higher than CT radiomics method as mentioned above (0.67 vs. 0.61), suggesting that CNN had better classification performance.

^18^F-FDG PET has been clinically recognized as a method to identify malignant solitary pulmonary nodules (SPN). Under the diagnostic criteria of maximum standard uptake value (SUV_max_) > 2.5, the sensitivity and specificity of ^18^F-FDG PET are 79–100% and 60–100%, respectively [40]. However, the diagnostic value of PET for GGN is still controversial. Many studies reported that GGNs were different from solid nodules, and PET imaging had a higher false-negative rate and false-positive rate for GGNs [41]. In this study, the classification performance of PET network was better than CT network, which seems to contradict the higher spatial resolution of CT. The latest study on identifying benign and malignant SPNs also found that the models based on PET radiomics features were generally better than those based on CT characteristics [19], consistent with previously published results [42–44]. These findings are interesting because they demonstrate that using PET texture features to assess radiotracer uptake's heterogeneity (discussed in [45, 46]) is as important as using CT texture features to analyze tissue density, although most literature is focused on CT.

Another possible factor limiting the accuracy of CNN is the use of data from only one imaging modality (PET or CT), which is an attempt to simplify and standardize data extraction [25]. The various machine learning techniques that have been studied (including CNN) have not yet produced predictions that can change clinical practice. In this study, we combined the data from PET and CT images, used these data to train a dual-stream CNN, and evaluated and analyzed the model performance [24]. Although PET/CT's performance was almost the same as PET network in the training set, its main performance indicators (accuracy, sensitivity, and NPV) were still higher than both CT and PET networks in the testing set. We confirmed PET/CT dual-mode imaging's advantages compared to conventional single-mode imaging from the perspective of multimodal deep learning. Besides, in this study, we only used a limited testing set (*n * = 33) to compare and evaluate the performance of PET/CT network and two nuclear medicine physicians. If our PET/CT network is robust in future tests with larger and more diverse data sets, this study may have a major clinical impact on assisting nuclear medicine physicians in making decisions about GGN detection and diagnosis.

Our study still had some limitations: (1) Although we used a data augmentation strategy in the training data, single-center research, small sample size, and imbalance between benign and malignant samples still affected CNN's performance. Specifically, three networks' performance (especially specificity) declined in the testing set compared to the training set, indicating overfitting. The solution is to continue to collect samples to increase the size of training data or to adopt multicenter research to improve the robustness of the network. (2) To ensure the sample size of CT data, we used 3-mm conventional CT instead of 1-mm HRCT (better detail performance), which may affect the performance of CT network. (3) During the screening of PET data, we removed a small number of IAC nodules that could not be segmented due to low PET uptake. For these GGNs, ^18^F-FDG is not applicable, and we look forward to developing a new PET imaging agent. (4) Also, our network was not "smart" enough. Although both CT and PET images use semiautomatic segmentation methods, there were still too many manual manipulations during the image preprocessing process. In the future, CNN should be used for image segmentation to truly achieve an "end-to-end" workflow.

## Conclusions

This study successfully constructed, trained, validated, and tested a dual-stream 3D-CNN based on ^18^F-FDG PET/CT and used it to classify benign lesions and IAC in GGNs. The dual-stream network was better than the CNN based on CT or PET alone. Therefore, in the absence of well-trained and experienced physicians, this CNN may help the differentiation and clinical management of benign and malignant GGNs. Besides, we also expect clinicians to use this CNN for other tumor research suitable for PET/CT imaging.

## Supplementary Information

**Additional file 1**. Report on image processing. Propensity score parameter list. Tumor segmentation. Training of the deep learning model

## Data Availability

The data supporting our findings are available upon request.

## Abbreviations

LDCT: Low-dose CT
GGN: Ground-glass opacity nodule
NSCLC: Non-small cell lung cancer
CNN: Convolutional neural networks
3D-CNN: Three-dimensional convolutional neural networks
IAC: Invasive adenocarcinoma
AAH: Atypical adenoma hyperplasia
AIS: Adenocarcinoma in situ
MIA: Microinvasive adenocarcinoma
IBSI: Imaging biomarker standardization initiative
EANM: European Association for Nuclear Medicine
HU: Hounsfield units
PSM: Propensity score matching
AUC: Area under the receiver operating characteristic curve
SPN: Solitary pulmonary nodules
SUV_max_: Maximum standard uptake value
PPV: Positive predictive value
NPV: Negative predictive value
