# Supplementary material for: Application of dual-stream 3D convolutional neural network based on 18F-FDG PET/CT in distinguishing benign and invasive adenocarcinoma in ground-glass lung nodules
Source: EJNMMI Phys. 2021 Nov 2;8:74. doi: 10.1186/s40658-021-00423-1 (PMC8561359; doi:10.1186/s40658-021-00423-1)
Supplement: Supplementary file 1 — Additional file 1. Report on image processing. Propensity score parameter list. Tumor segmentation. Training of the deep learning model [file 40658_2021_423_MOESM1_ESM.docx]

**Supplementary material–**Report on image processing

| Acquisition and reconstruction | \| **Acquisition parameters** \| **Siemens Biograph mCT 64** \| \| \| --- \| --- \| --- \| \| **PET** \| **CT** \| \| **^18^F-FDG activity (MBq)*** \| 350-550 (3.70-7.77MBq/kg) \| – \| \| **Min/bed position** \| 2.5 \| – \| \| **Crystal** \| LSO \| – \| \| **Reconstruction** \| OSEM + PSF + TOF \| – \| \| **Matrix (pixels)** \| 200×200 \| 512×512 \| \| **Resolution (mm)** \| 4.0 \| 0.7 \| \| **Slice thickness (mm)** \| 3mm \| 3mm \| \| **Slices** \| – \| 64 \| \| **Voltage (kV)** \| – \| 140 \| \| **Tube current (mA)** \| – \| 64 \| \| **Reconstruction** \| Gaussian filtering with a full-width at half maximum of 2.0mm; 2 iterations and 21 subsets \| B70f very sharp \| \| *Administered activity was calculated according to the European Association of Nuclear Medicine (EANM) guidelines, version 1.0, and from February 2015, version 2.0 \| \| \| |
| --- | --- | --- | --- | --- | --- | --- | --- | --- | --- | --- | --- | --- | --- | --- | --- | --- | --- | --- | --- | --- | --- | --- | --- | --- | --- | --- | --- | --- | --- | --- | --- | --- | --- | --- | --- | --- | --- | --- | --- | --- | --- | --- |
| Approach | The images were analyzed as a volume (3D). |
| Process structure | Image acquisition -> reconstruction ->anonymization -> segmentation -> export -> data augmentation -> CNN training and testing |
| Software | 3D-Slicer 4.11.20200930 (www.slicer.com)  Python 3.8.5 (www.python.org)  Tensorflow-gpu 2.3.1 (Google, www.tensorflow.org) |
| Data availability | All the original patient DICOM files are stored in the institutional PACS. Anonymized DICOM files are stored on the Department's hard disk. The trained models are stored in the local server. |

| **Data conversion** | |
| --- | --- |
| Procedure | None |
| **Image post-acquisition processing** | |
| Procedure | None |
| **Segmentation** | |
| ROI | The VOI included the primary tumor lesion. To consider the tumor edge pattern, the generated bounding box is dilated by 1 pixel (PET images) and 3.00mm (CT images). |
| Procedure | The VOIs were semi-automatically defined on PET images using a semi-automatic segmentation method developed by Beichel et al. [[1](#_ENREF_1)] (3D-Slicer, PET-IndiC extension). VOIs on CT images were semi-automatically defined with NVIDIA AI-Assisted Annotation (3D-Slicer, Segment Editor module), using boundary point-based CT segmentation model of lung tumor. |
| **Interpolation** |  |
| Voxel dimensions | 4.07mm^3^ for PET images; 0.73mm^3^ for CT images |
| Image interpolation method | Nearest Neighbor |
| Image intensity rounding | Not applicable |
| ROI interpolation method | Not applicable |
| ROI partial volume | Not applicable |
| **Re-segmentation** |  |
| ROI mask criteria | None |
| **Discretization** |  |
| Discretization method | None |
| **Feature calculation** |  |
| Feature set | None |
| Feature parameters | None |

**Supplementary material–**Propensity score parameter list

| The variables used in calculating the propensity score | Age, gender, smoking history, fasting blood glucose, GGN number grouping |
| --- | --- |
| Propensity score algorithm | Logistic regression model |
| C-statistical | 0.6864 |
| Matching method | Greed matching within specified caliper distances |
| Distance metric | 0.6 |
| Matching ratio | 4:1 |
| Use of replacement | With replacement |
| Matching sample size | No Y=0: 92 cases Total:115 |
|  | No Y=1: 23 cases |

**Supplementary material–**Tumor segmentation


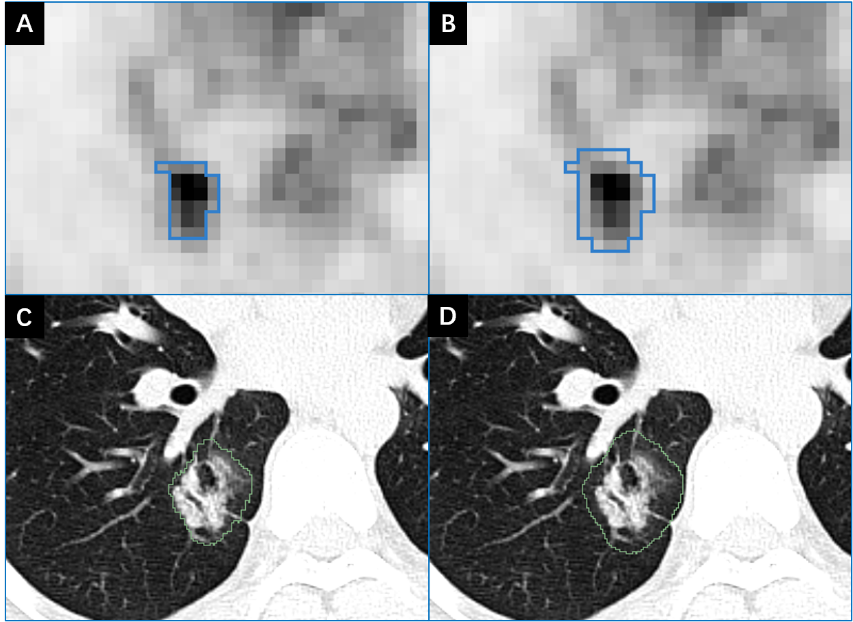


**Figure S1. 42-year-old man with mixed ground-glass opacity nodule (GGN). A-B： the PET image was semi-automatically segmented, and the generated boundary was dilated by 1 pixel. C-D: the CT image was semi-automatically segmented, and the generated boundary was dilated by 3.00mm.**

**Supplementary material–**Training of the deep learning model


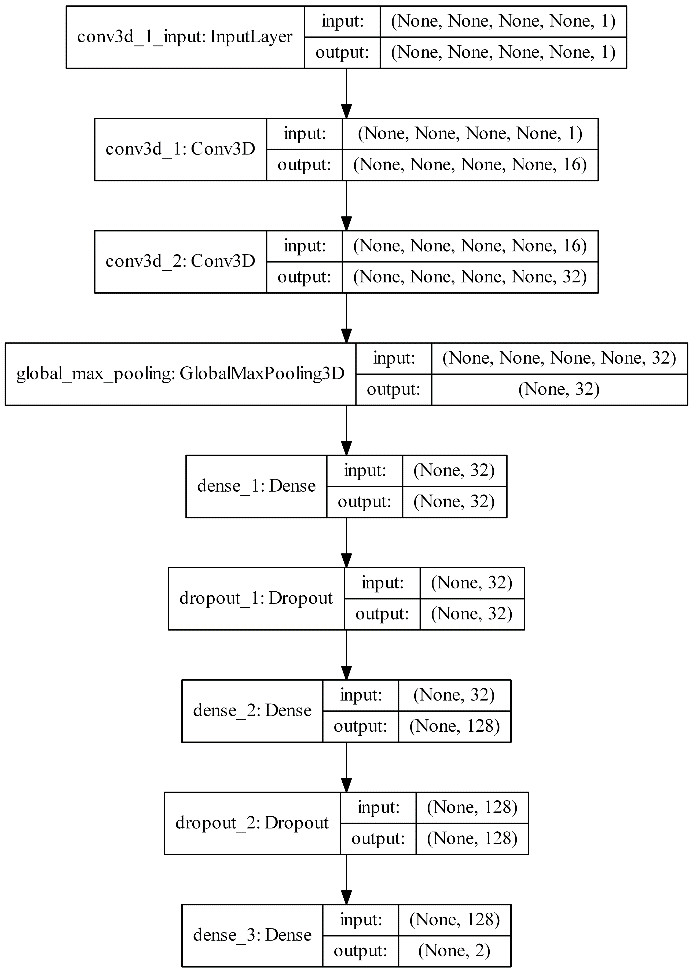


**Figure S2. Tumor size adaptive PET 3D-CNN**

More specifically, two 3D convolutional layers with 16 and 32 convolution kernels (5×5×5) are followed by the global max pooling layer [[2](#_ENREF_2)]. The activation function of the two convolutional layers is tanh. Regardless of the input size, the global max pooling layer will generate a 32-dimensional feature vector. Besides, the fully connected layer produces a 128-dimensional feature vector. Implement a dropout of 0.5 after the fully connected layer to avoid overfitting [[3](#_ENREF_3)]. The target output is whether GGN is benign or IAC. Here, we tried the optional global average pooling layer and found that the model's fitting effect became worse.


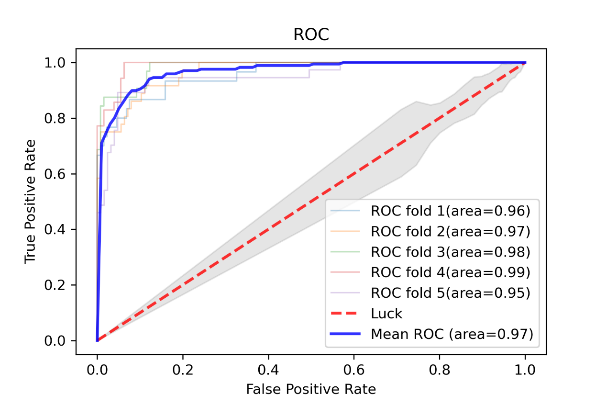

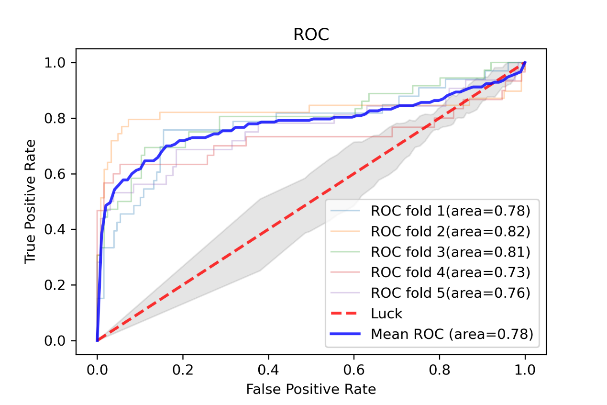


**Figure S3. Comparison of the training effects of PET-CNN global max pooling and global average pooling**


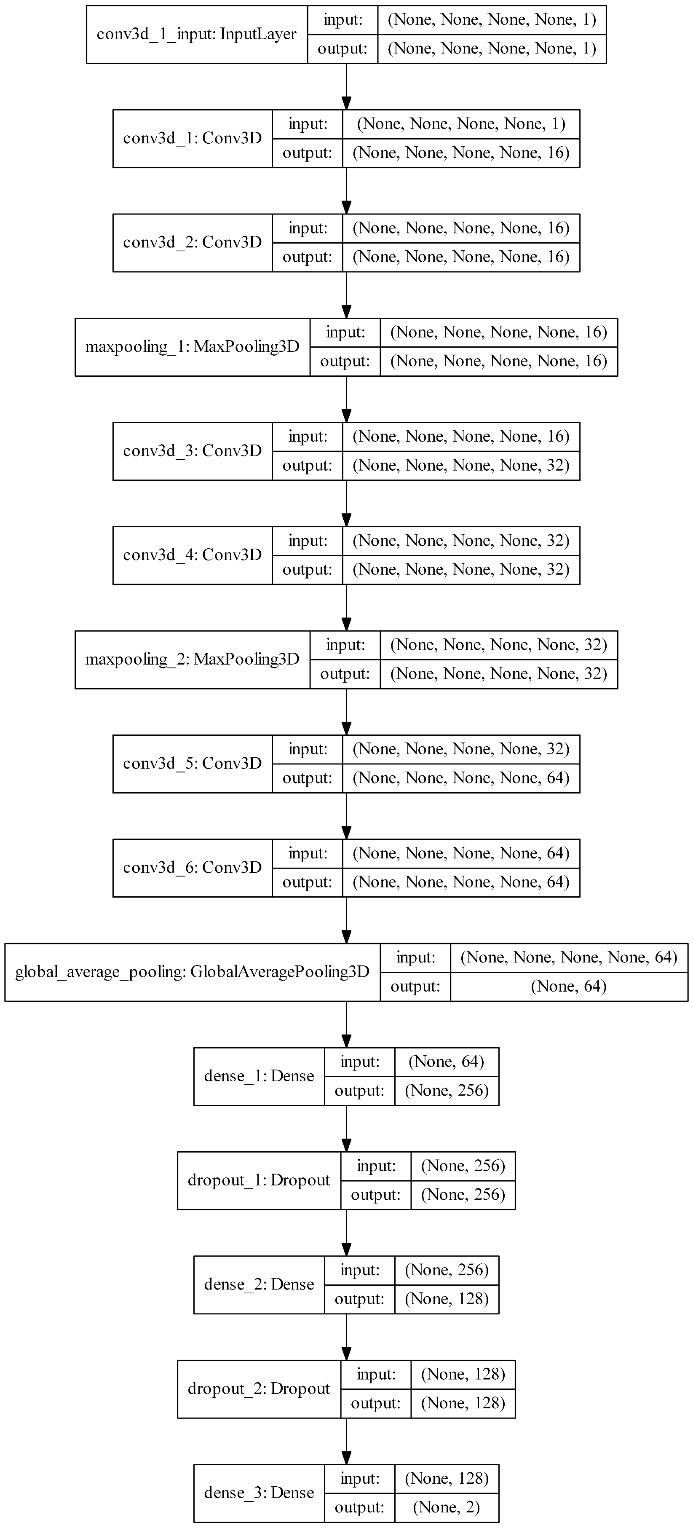


**Figure S4. Tumor size adaptive CT 3D-CNN**

Considering the smaller pixels and larger matrix of CT, we adopted a deeper CNN and a larger network. More specifically, two 3D convolutional layers with 16 convolution kernels (5×5×5) are followed by the maxpooling layer, followed by two 3D convolutional layers of 32 convolution kernels (5×5×5) and maxpooling layer, followed by two 3D convolutional layers of 64 convolution kernels (5×5×5) and global average pooling layer [[4](#_ENREF_4)]. The activation function of the convolutional layer is tanh. Regardless of the input size, the global average pooling layer will generate a 64-dimensional feature vector. Besides, the fully connected layer produces a 128-dimensional feature vector. Implement a dropout of 0.5 after the fully connected layer to avoid overfitting [[3](#_ENREF_3)]. The target output is whether GGN is benign or IAC. Here, we tried the optional global max pooling layer and found that the training process model does not converge.


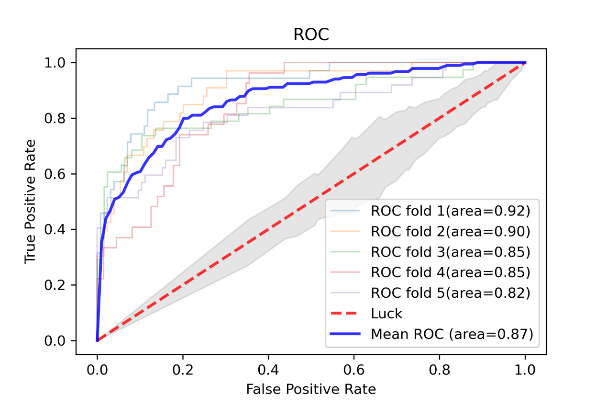

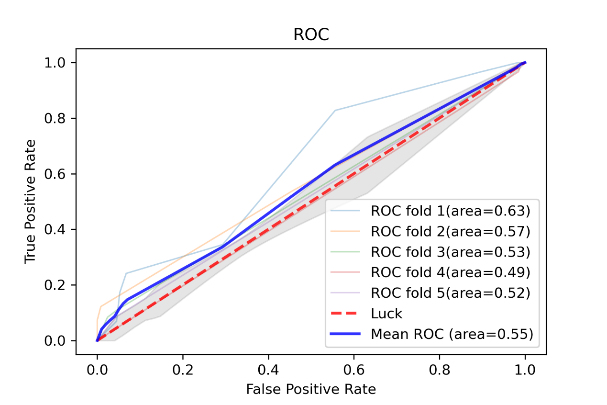


**Figure S5. Comparison of the training effects of CT-CNN global average pooling and global max pooling**


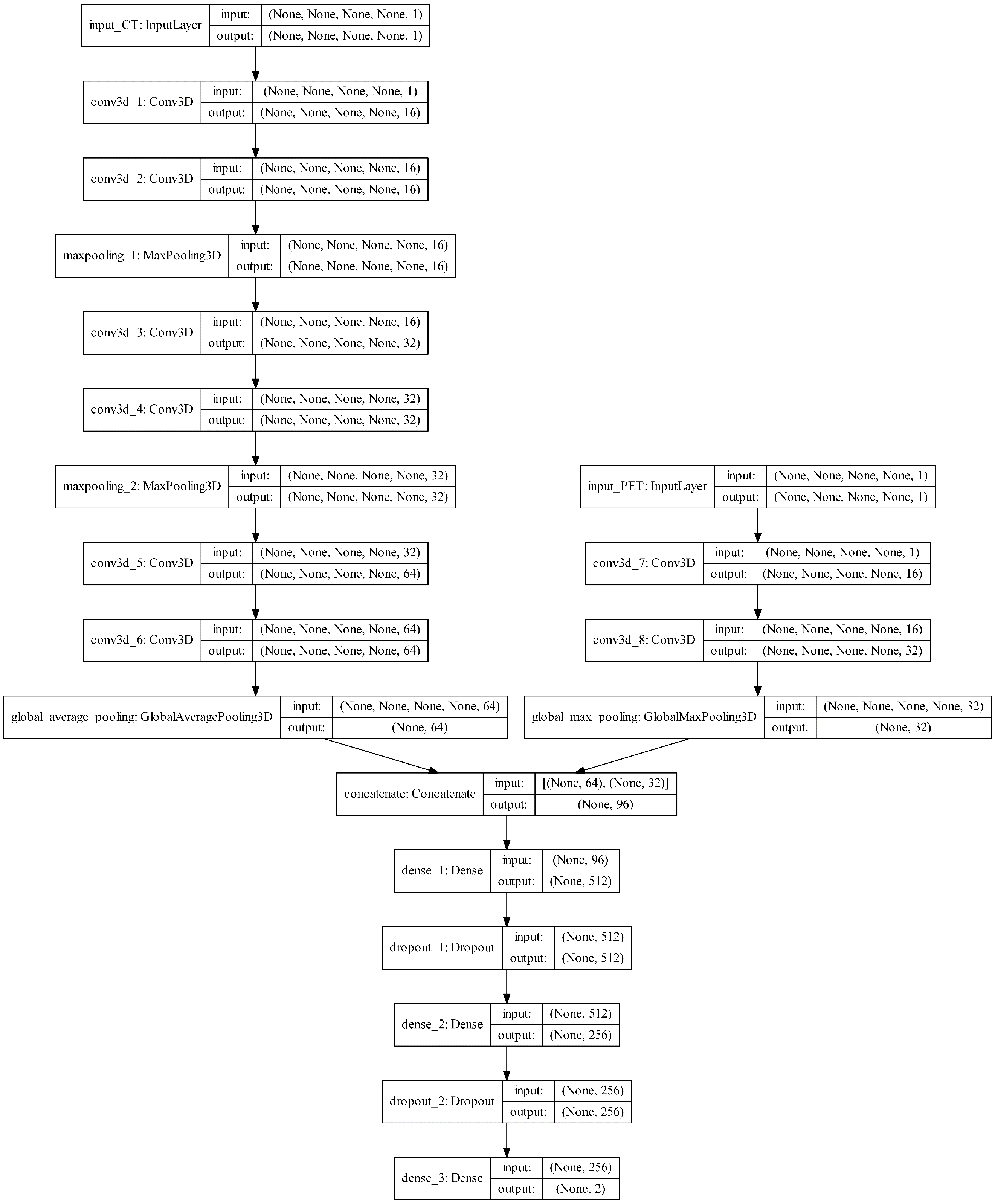


**Figure S6. Tumor size adaptive PET/CT 3D-CNN**

To integrate PET and CT information, we connect the two inputs of PET and CT through the concatenate layer to generate a 96-dimensional feature vector. Besides, the fully connected layer produces a 256-dimensional feature vector. Implement a dropout of 0.5 after the fully connected layer to avoid overfitting [[3](#_ENREF_3)]. The target output is whether GGN is benign or IAC.

**References**

1. Beichel RR, Van Tol M, Ulrich EJ, Bauer C, Chang T, Plichta KA, et al. Semiautomated segmentation of head and neck cancers in 18F-FDG PET scans: A just-enough-interaction approach. Medical physics. 2016;43:2948-64. doi:10.1118/1.4948679.

2. Zhou B, Khosla A, Lapedriza A, Oliva A, Torralba A. Learning deep features for discriminative localization. Proceedings of the IEEE conference on computer vision and pattern recognition; 2016. p. 2921-9.

3. Srivastava N, Hinton G, Krizhevsky A, Sutskever I, Salakhutdinov R. Dropout: a simple way to prevent neural networks from overfitting. The journal of machine learning research. 2014;15:1929-58.

4. Lin M, Chen Q, Yan S. Network in network. arXiv preprint arXiv:13124400. 2013.
